# Supporting plots and tables on vapour–liquid equilibrium prediction for synthesis gas conversion using artificial neural networks

**DOI:** 10.1016/j.dib.2018.10.129

**Published:** 2018-10-29

**Authors:** Precious Chukwuweike Eze, Cornelius Mduduzi Masuku

**Affiliations:** Department of Civil and Chemical Engineering, University of South Africa, Private Bag X6, Florida, 1710, South Africa

**Keywords:** Artificial neural networks, Fischer–Tropsch reaction, Machine learning, Thermodynamic modeling, Phase equilibrium

## Abstract

This article contains data on vapor–liquid equilibrium modeling of 1533 gas-liquid solubilities divided over sixty binary systems viz. carbon monoxide, carbon dioxide, hydrogen, water, ethane, propane, pentane, hexane, methanol, ethanol, 1-propanol, 1-butanol, 1-pentanol, and 1-hexanol in the solvents phenanthrene, 1-hexadecanol, octacosane, hexadecane and tetraethylene glycol at pressures up to 5.5 MPa and temperatures from 293 to 553 K using literature data. The solvents are considered to be potentially significant in the conversion of synthesis gas through gas-slurry processes. Artificial neural networks limited to one hidden layer and up to five neurons in the hidden layer were used to predict the binary plots.

**Specifications table**

**Table:** 

Subject area	*Chemical Engineering*
More specific subject area	*Thermodynamic Phase Equilibria*
Type of data	*Tables, and Figures*
How data was acquired	*Generated through a code on MATLAB*^*®*^
Data format	*Filtered*
Experimental factors	*Feature scaling*
Experimental features	*Code was implemented on MATLAB*^*®*^
Data source location	*Input experimental data was obtained from*Breman et al. (1994).
Data accessibility	*Source data is available on Journal of Chemical & Engineering Data:*https://pubs.acs.org/doi/abs/10.1021/je00016a004
Related research article	P.C. Eze, C.M. Masuku, Vapour–Liquid Equilibrium Prediction for Synthesis Gas Conversion using Artificial Neural Networks, *SAJCE* (2018) in press.

**Value of the data**

• This data could be used by the broader scientific community as it shows the training and testing of artificial neural networks for a number of binary systems.
• Different training algorithms could be used and compared with the performance described here.
• Other computational methods and techniques could be used and compared with the data presented here.

## 1 Data

The data presented here is generated in preparation of a manuscript on vapour–liquid equilibrium (VLE) prediction for synthesis gas conversion using artificial neural networks (ANN) [1]. The experimental VLE data used in this study was obtained from Breman et al. [2]. Phase equilibrium modeling is a crucial element in describing the behavior of the Fischer–Tropsch (FT) reaction [3], [4], [5], [6], [7], [8], [9], [10], [11], [12]. The FT reaction produces a range of hydrocarbons from light olefins and paraffins to heavy wax. Since we are comparing too many binaries to easily visualize, data for each binary is presented here. Then the summary of the overall results is published on the related paper [1]. The tables and figures presented here are unique; there is no duplication.

## 2 Experimental design, materials, and methods

An artificial neural network with input, hidden, and output layers was generated. The network was limited to one hidden layer and a maximum of five neurons in the hidden layer. A small network with the required accuracy is desirable for the speed of computation.

**Fig. 1:**
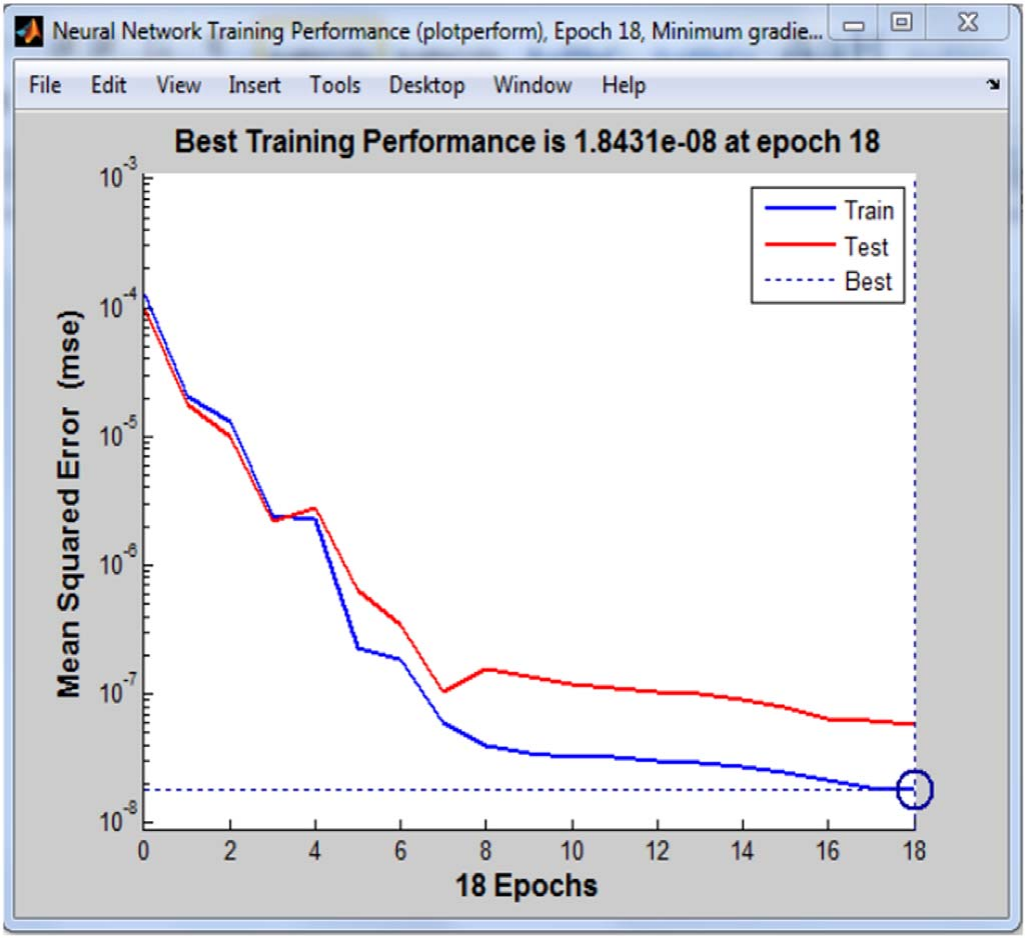
Neural network performance plot.

To validate the networks, the performance plots were generated for all the binary systems. The training and test curves for one representative system is presented in Fig. 1.

The performance plot does not indicate any major problems with the training. The training and test curves are very similar. If the test curves had increased significantly before the training curve increased, then it is possible that some overfitting might have occurred. The best training performance which is represented by the property tr.best_epoch indicates the iteration at which the validation performance reached a minimum. The training continued for 18 more iteration before the training stopped.

The next step is to evaluate the training state plot. The training record is used to plot the training state plot.

**Fig. 2:**
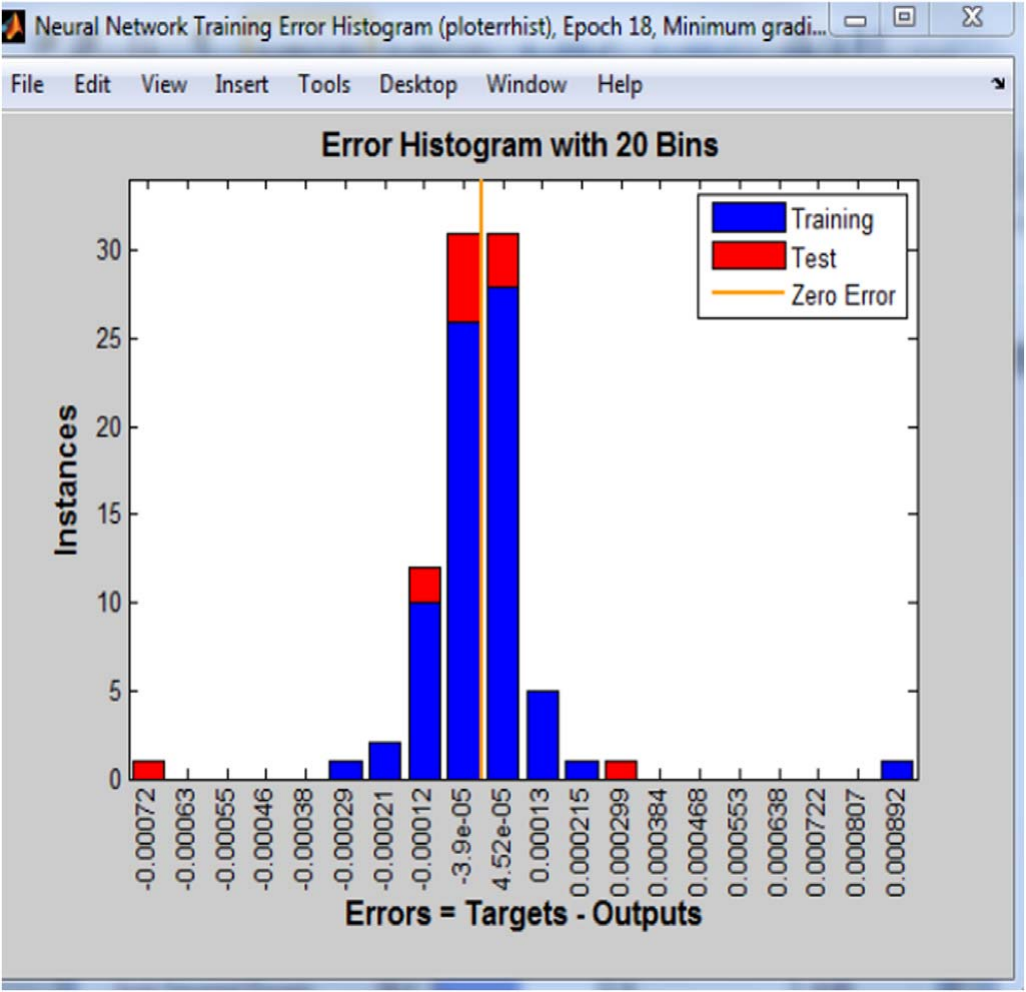
Neural network error histogram.

Another plot used to validate the network performance is the error histogram presented in Fig. 2. The error histogram plots a histogram of error values. It computes the error values as the difference between target values and predicted values, helping us to visualize the networks error.

**Fig. 3:**
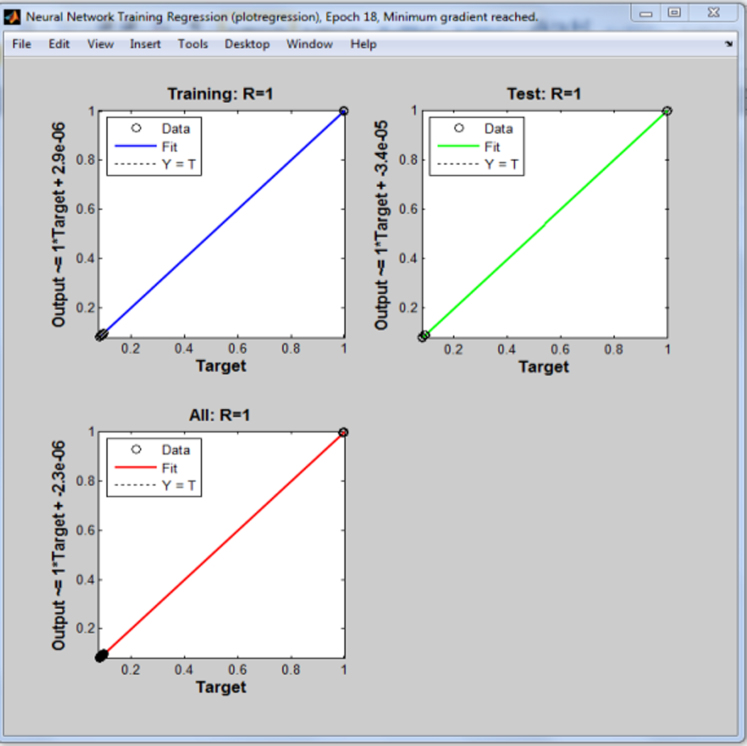
Neural network regression plot.

The low values in the error histogram are an indication of a good network performance. The final step in validating the network results is by plotting a regression plot shown in Fig. 3. The solid line in the plot represents the best linear fit regression between outputs and targets.

From Fig. 3, it can be observed that training was perfect. The *R* value of 1 for the training and test data indicates that there is an exact linear relationship between our inputs and targets.

It is important to note that the plots in Fig. 1, Fig. 2, Fig. 3 were obtained after training one of the binary system, and is used to represent the plots obtained when training the entire binary system since similar plots were also obtained for each binary system.

The percentage MAE, and RMSE across each system are presented in Table 1.

**Table 1:** %MAE and %RMSE values across each binary system.

no.	**Solute**	**Solute**	**%MAE X**_**i**_	**%MAE Y**_**i**_	**%RMSE X**_**i**_	**%RMSE Y**_**i**_
1	**CO**_**2**_	**C**_**8**_**H**_**18**_**O**_**5**_	−0.0020	0.0008	0.0210	0.0250
2	**CO**	**C**_**8**_**H**_**18**_**O**_**5**_	−0.0030	−0.0102	0.0560	0.0901
3	**H**_**2**_	**C**_**8**_**H**_**18**_**O**_**5**_	0.0000	−0.0039	0.0150	0.0451
4	**CH**_**3**_**OH**	**C**_**8**_**H**_**18**_**O**_**5**_	−0.0260	−0.0321	0.1120	0.1235
5	**C**_**2**_**H**_**5**_**OH**	**C**_**8**_**H**_**18**_**O**_**5**_	0.0020	−0.0005	0.0150	0.0093
6	**C**_**3**_**H**_**7**_**OH**	**C**_**8**_**H**_**18**_**O**_**5**_	0.0007	−0.0038	0.0090	0.0154
7	**C**_**4**_**H**_**9**_**OH**	**C**_**8**_**H**_**18**_**O**_**5**_	−0.0010	−0.0005	0.0040	0.0051
8	**C**_**5**_**H**_**11**_**OH**	**C**_**8**_**H**_**18**_**O**_**5**_	0.0000	−0.0105	0.0080	0.1979
9	**H**_**2**_**O**	**C**_**8**_**H**_**18**_**O**_**5**_	0.0019	0.0001	0.0090	0.0105
10	**CO**_**2**_	**C**_**10**_**H**_**14**_	0.0022	−0.0004	0.0110	0.0091
11	**CO**	**C**_**10**_**H**_**14**_	−0.0001	0.0007	0.0070	0.0071
12	**H**_**2**_	**C**_**10**_**H**_**14**_	0.0005	−0.0005	0.0070	0.0068
13	**CH**_**3**_**OH**	**C**_**10**_**H**_**14**_	0.0012	−0.0031	0.0040	0.0063
14	**C**_**2**_**H**_**5**_**OH**	**C**_**10**_**H**_**14**_	0.0013	−0.0017	0.0070	0.0108
15	**C**_**3**_**H**_**7**_**OH**	**C**_**10**_**H**_**14**_	0.0006	−0.0020	0.0050	0.0091
16	**C**_**4**_**H**_**9**_**OH**	**C**_**10**_**H**_**14**_	−0.0020	−0.0005	0.0210	0.0160
17	**C**_**5**_**H**_**11**_**OH**	**C**_**10**_**H**_**14**_	0.0009	−0.0014	0.0090	0.0136
18	**H**_**2**_**O**	**C**_**10**_**H**_**14**_	−0.0050	−0.0026	0.0100	0.0090
19	**CO**	**C**_**16**_**H**_**34**_	−0.0008	−0.0039	0.0100	0.0158
20	**H**_**2**_	**C**_**16**_**H**_**34**_	−0.0010	−0.0019	0.0310	0.0141
21	**CO**_**2**_	**C**_**16**_**H**_**34**_	0.0003	0.0002	0.0100	0.0054
22	**C**_**2**_**H**_**6**_	**C**_**16**_**H**_**34**_	0.0002	−0.0001	0.0000	0.0052
23	**C**_**3**_**H**_**8**_	**C**_**16**_**H**_**34**_	0.0005	−0.0001	0.0030	0.0027
24	**C**_**5**_**H**_**12**_	**C**_**16**_**H**_**34**_	−0.0008	0.0002	0.0030	0.0043
25	**C**_**6**_**H**_**14**_	**C**_**16**_**H**_**34**_	−0.0002	−0.0003	0.0040	0.0051
26	**CH**_**3**_**OH**	**C**_**16**_**H**_**34**_	−0.0001	−0.2533	10.030	0.4175
27	**C**_**2**_**H**_**5**_**OH**	**C**_**16**_**H**_**34**_	−0.0009	0.0009	0.0160	0.0142
28	**C**_**3**_**H**_**7**_**OH**	**C**_**16**_**H**_**34**_	0.0034	−0.0077	0.0430	0.0294
29	**C**_**4**_**H**_**9**_**OH**	**C**_**16**_**H**_**34**_	−0.0030	−0.0085	0.0110	0.0193
30	**C**_**5**_**H**_**11**_**OH**	**C**_**16**_**H**_**34**_	0.0008	−0.0014	0.0060	0.0056
31	**C**_**6**_**H**_**13**_**OH**	**C**_**16**_**H**_**34**_	−0.0030	−0.0008	0.0140	0.0091
32	**H**_**2**_**O**	**C**_**16**_**H**_**34**_	0.0032	−0.0172	0.0790	0.1900
33	**CO**	**C**_**16**_**H**_**33**_**OH**	0.0082	−0.0005	0.0490	0.0071
34	**CO**_**2**_	**C**_**16**_**H**_**33**_**OH**	0.0034	−0.0001	0.0260	0.0079
35	**H**_**2**_	**C**_**16**_**H**_**33**_**OH**	−0.0010	0.0055	0.0190	0.0507
36	**C**_**2**_**H**_**6**_	**C**_**16**_**H**_**33**_**OH**	−0.0050	0.0423	0.0380	0.2254
37	**C**_**3**_**H**_**8**_	**C**_**16**_**H**_**33**_**OH**	0.0038	−0.0126	0.0090	0.0691
38	**C**_**5**_**H**_**12**_	**C**_**16**_**H**_**33**_**OH**	0.0012	0.0004	0.0260	0.0123
39	**C**_**6**_**H**_**14**_	**C**_**16**_**H**_**33**_**OH**	0.0107	0.0157	0.0870	0.2889
40	**CH**_**3**_**OH**	**C**_**16**_**H**_**33**_**OH**	−0.0009	0.0001	0.0130	0.0078
41	**C**_**2**_**H**_**5**_**OH**	**C**_**16**_**H**_**33**_**OH**	0.0068	−0.0123	0.0740	0.2276
42	**C**_**3**_**H**_**7**_**OH**	**C**_**16**_**H**_**33**_**OH**	0.0007	−0.0001	0.0050	0.0146
43	**C**_**4**_**H**_**9**_**OH**	**C**_**16**_**H**_**33**_**OH**	0.0048	−0.0055	0.0170	0.0188
44	**C**_**5**_**H**_**11**_**OH**	**C**_**16**_**H**_**33**_**OH**	0.0005	0.0006	0.0040	0.0125
45	**C**_**6**_**H**_**13**_**OH**	**C**_**16**_**H**_**33**_**OH**	−0.0090	−0.0287	0.0140	0.0463
46	**H**_**2**_**O**	**C**_**16**_**H**_**33**_**OH**	−0.0270	0.0607	0.1210	0.1731
47	**CO**	**C**_**28**_**H**_**58**_	−0.1100	0.0000	0.4810	0.0000
48	**H**_**2**_	**C**_**28**_**H**_**58**_	0.0343	0.0000	0.2100	0.0000
49	**CO**_**2**_	**C**_**28**_**H**_**58**_	0.1270	0.0000	0.5600	0.0000
50	**C**_**2**_**H**_**6**_	**C**_**28**_**H**_**58**_	0.0059	0.0000	0.0880	0.0000
51	**C**_**3**_**H**_**8**_	**C**_**28**_**H**_**58**_	0.0055	0.0000	0.0180	0.0000
52	**C**_**5**_**H**_**12**_	**C**_**28**_**H**_**58**_	0.0039	0.0000	0.0150	0.0000
53	**C**_**6**_**H**_**14**_	**C**_**28**_**H**_**58**_	0.0018	0.0000	0.0170	0.0000
54	**CH**_**3**_**OH**	**C**_**28**_**H**_**58**_	−0.0040	0.0000	0.0200	0.0000
55	**C**_**2**_**H**_**5**_**OH**	**C**_**28**_**H**_**58**_	−0.0110	0.0000	0.0300	0.0000
56	**C**_**3**_**H**_**7**_**OH**	**C**_**28**_**H**_**58**_	0.0042	0.0000	0.1870	0.0000
57	**C**_**4**_**H**_**9**_**OH**	**C**_**28**_**H**_**58**_	−0.0030	0.0000	0.0300	0.0000
58	**C**_**5**_**H**_**11**_**OH**	**C**_**28**_**H**_**58**_	0.0059	0.0000	0.0150	0.0000
59	**C**_**6**_**H**_**13**_**OH**	**C**_**28**_**H**_**58**_	0.0002	0.0000	0.0170	0.0000
60	**H**_**2**_**O**	**C**_**28**_**H**_**58**_	−0.0150	0.0000	0.0340	0.0000

The experimental values versus the predicted values for the *X*_*i*_ and *Y*_*i*_ for all the 60 binaries denoted (A1–A60) are presented in Fig. 4, Fig. 5, Fig. 6, Fig. 7, Fig. 8, Fig. 9, Fig. 10, Fig. 11, Fig. 12, Fig. 13, Fig. 14, Fig. 15.

**Fig. 4:**
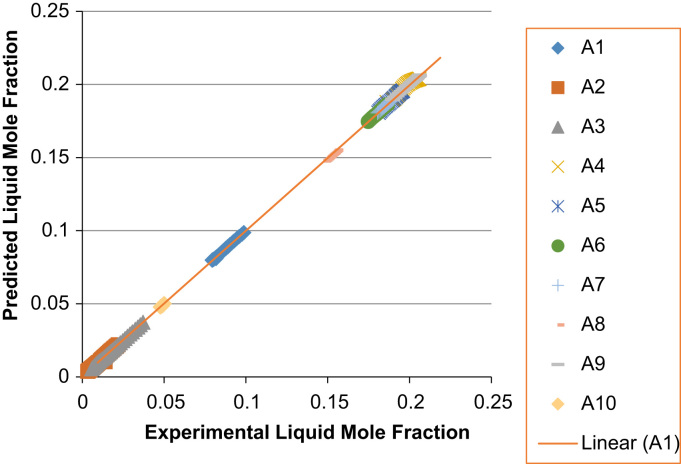
Experimental Liquid Mole Fraction versus Predicted Liquid Mole fraction for system A1–A10.

**Fig. 5:**
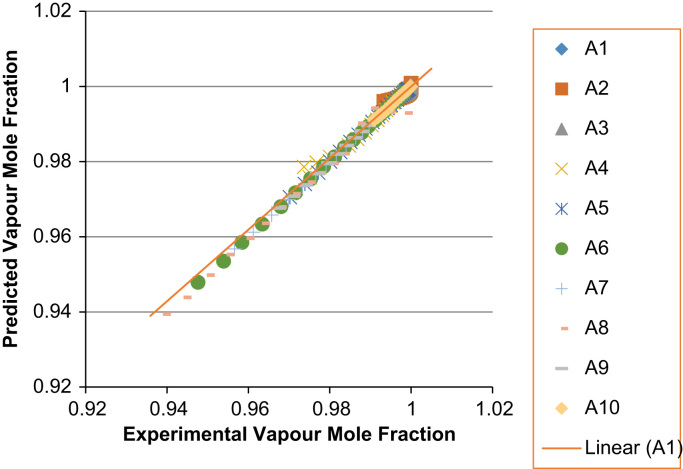
Experimental Vapor Mole Fraction versus Predicted Vapor Mole fraction for system A1–A10.

**Fig. 6:**
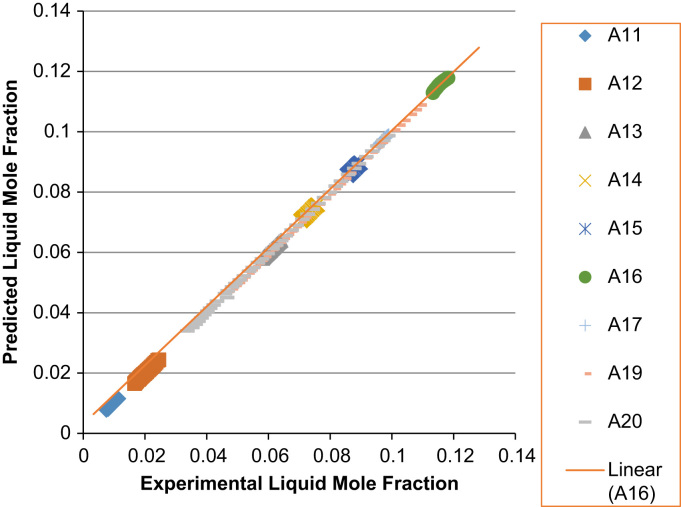
Experimental Liquid Mole Fraction versus Predicted Liquid Mole fraction for system A11–A20.

**Fig. 7:**
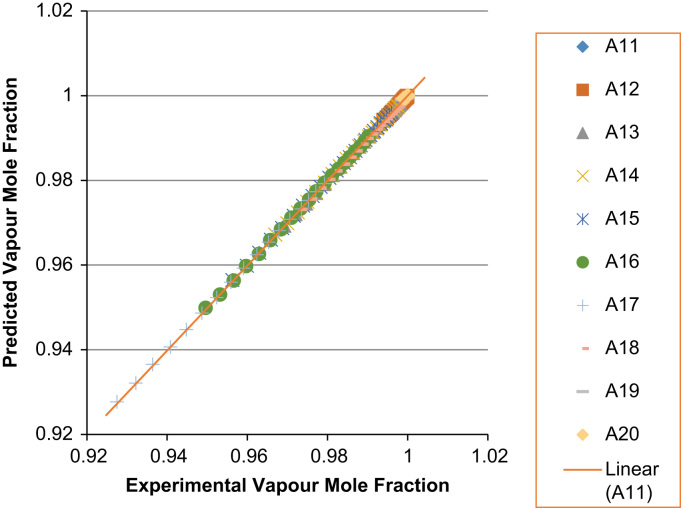
Experimental Vapor Mole Fraction versus Predicted Vapor Mole fraction for system A11–A20.

**Fig. 8:**
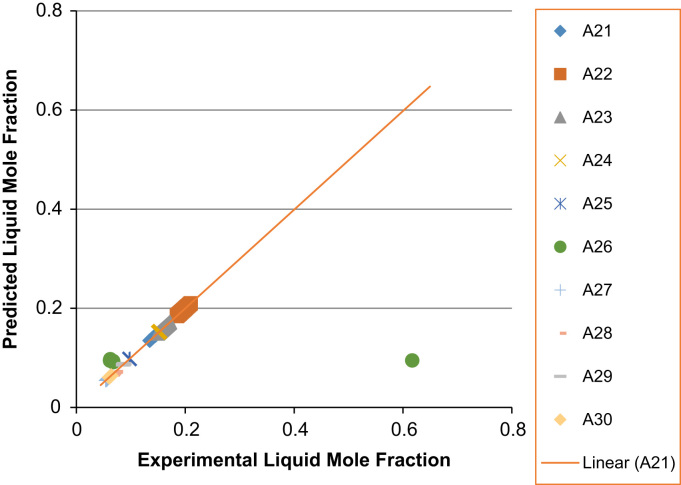
Experimental Liquid Mole Fraction versus Predicted Liquid Mole fraction for system A21–A30.

**Fig. 9:**
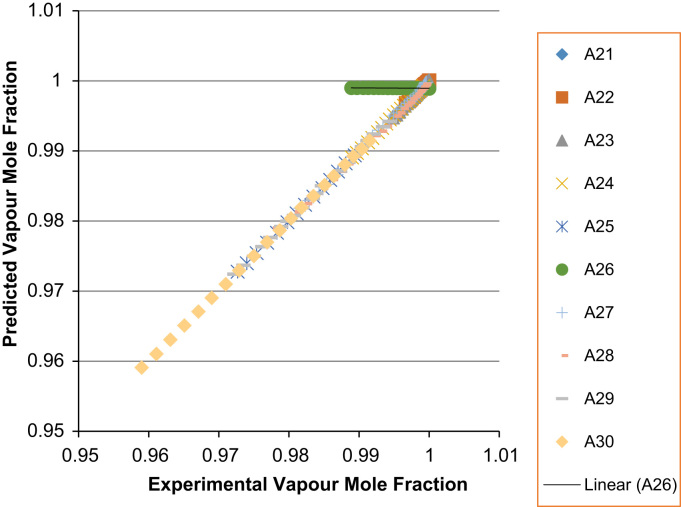
Experimental Vapor Mole Fraction versus Predicted Vapor Mole fraction for system A21–A30.

**Fig. 10:**
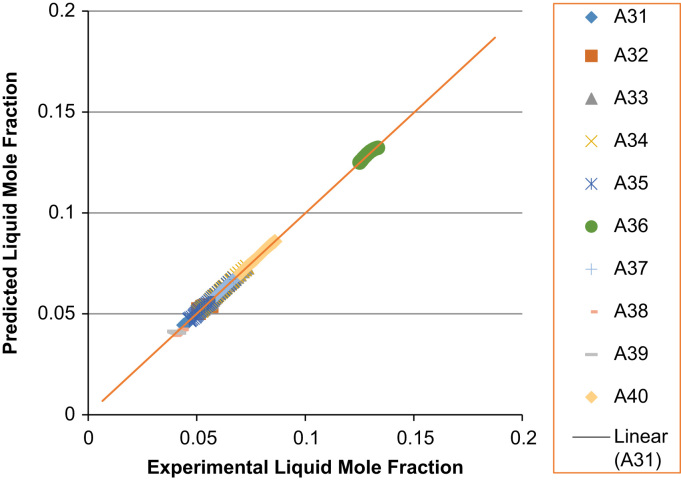
Experimental Liquid Mole Fraction versus Predicted Liquid Mole fraction for system A31–A40.

**Fig. 11:**
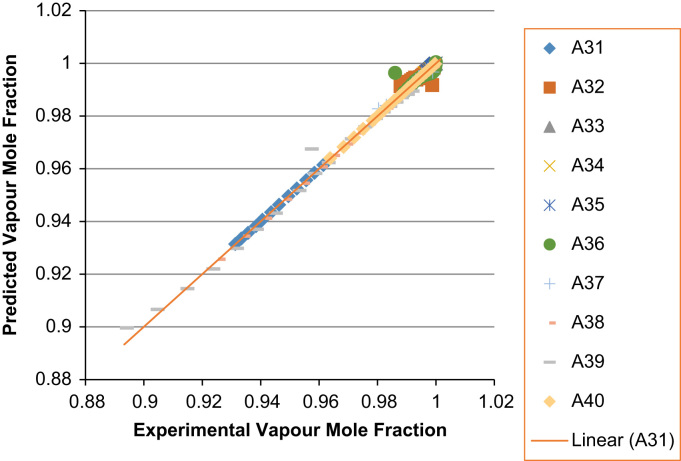
Experimental Vapor Mole Fraction versus Predicted Vapor Mole fraction for system A31–A40.

**Fig. 12:**
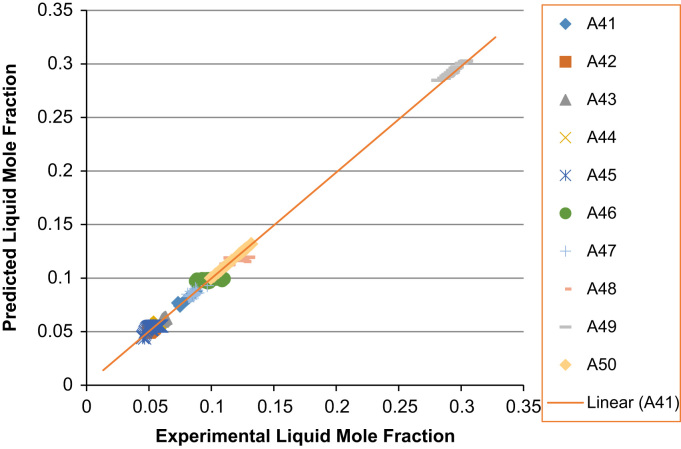
Experimental Liquid Mole Fraction versus Predicted Liquid Mole fraction for system A41–A50.

**Fig. 13:**
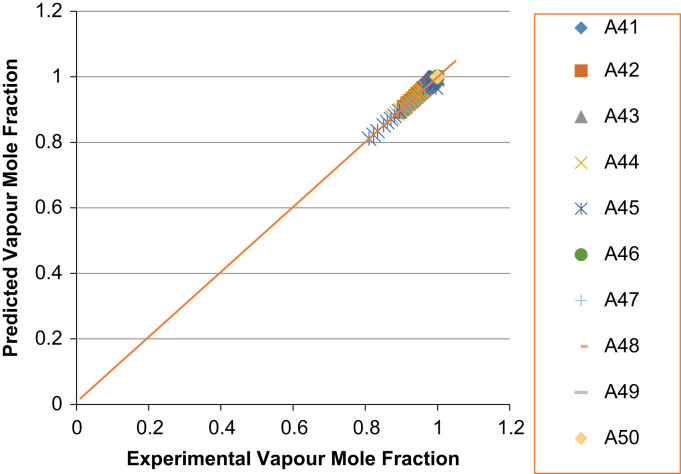
Experimental Vapor Mole Fraction versus Predicted Vapor Mole fraction for system A41–A50.

**Fig. 14:**
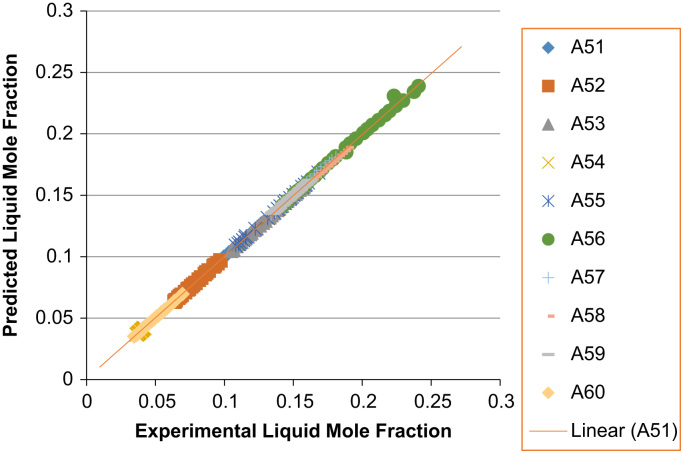
Experimental Liquid Mole Fraction versus Predicted Liquid Mole fraction for system A51–A60.

**Fig. 15:**
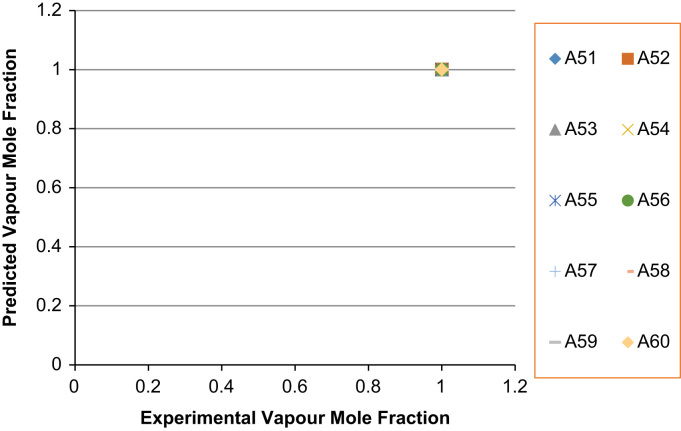
Experimental Vapor Mole Fraction versus Predicted Vapor Mole fraction for system A51–A60.
