# Supplementary material for: Supporting plots and tables on vapour–liquid equilibrium prediction for synthesis gas conversion using artificial neural networks
Source: Data Brief. 2018 Oct 29;21:1435–44. doi: 10.1016/j.dib.2018.10.129 (PMC6234271; doi:10.1016/j.dib.2018.10.129)
Supplement: Supplementary file 1 — Supplementary material [file mmc1.docx]

There are no competing interests to be declared.
